# The O28 Antigen Gene Clusters of *Salmonella enterica* subsp. *enterica* Serovar Dakar and Serovar Pomona Are Different

**DOI:** 10.1155/2010/209291

**Published:** 2010-06-28

**Authors:** Clifford G. Clark, Christopher C. R. Grant, Keri M. Trout-Yakel, Helen Tabor, Lai-King Ng, Kris Rahn, Kristyn Franklin, Andrew M. Kropinski

**Affiliations:** ^1^Enteric Diseases Program, Bacteriology and Enteric Diseases Program, National Microbiology Laboratory, Public Health Agency of Canada, Winnipeg, MB, Canada R3E 3R2; ^2^Department of Medical Microbiology, University of Manitoba, 510 Basic Medical Sciences Building, 730 William Aveue, Winnipeg, MB, Canada R3T 2N2; ^3^Laboratory for Foodborne Zoonoses, Public Health Agency of Canada, Guelph, ON, Canada N1G 3W4; ^4^Molecular and Cellular Biology, University of Guelph, Guelph, ON, Canada N1G 2W1

## Abstract

A 10 kb O-antigen gene cluster was sequenced from a *Salmonella enterica* subsp. *enterica* Dakar O28 reference strain and from two *S*. Pomona serogroup O28 isolates. The two *S*. Pomona O antigen gene clusters showed only moderate identity with the *S*. Dakar O28 gene cluster, suggesting that the O antigen oligosaccharides may contain one or more sugars conferring the O28 epitope but may otherwise be different. These novel findings are absolutely critical for the correct interpretation of molecular serotyping assays targeting genes within the O antigen gene clusters of these *Salmonella* serotypes and suggest the possibility that the O antigen gene clusters of other *Salmonella* serovars may also be heterogenous.

## 1. Introduction

*Salmonella *O serotyping utilizes antibodies specific for sugars in the lipopolysaccharide O antigen side chain to differentiate among serovars of this bacterium. Antibodies, in highly absorbed serotyping reagents, frequently recognize a very small epitope within the O antigen oligosaccharide, perhaps only one sugar or part of one sugar [1]. The O antigen of Gram-negative bacteria is a highly variable, surface-exposed component of the lipopolysaccharide which contributes a significant role to the cell surface antigenic variation of these bacteria. It consists of repeated 3–6 monosaccharide units, with the variation in O antigens due to differences in the composition of the monosaccharide units and sugar linkages. Variations among O antigens provide the structural basis for both the *Salmonella* Kauffmann-White [1] and *Escherichia coli* O antigen serotyping schemes [2]. There are currently 46 O antigens recognized in the 2541 serovars comprising *Salmonella *[3] and 174 types of O antigen in *E. coli *[4].

Serotype currently provides the baseline from which other typing methods are carried out [5, 6]. Both the virulence and host range of *Salmonella enterica* isolates are serotype specific [7, 8]; thus, accurate determination of *Salmonella* serotype is currently essential for human disease surveillance and outbreak detection [9] and control of the organism in the food chain [10]. Additionally, the serogroup classification as defined by the Kauffmann-White scheme often indicates genetic relatedness, indicating that serotype frequently has phylogenetic significance [11].

The complexity, cost, and time required for the traditional serotyping using custom or commercial antisera have led researchers to consider development of alternative molecular methods [12]. Consequently it is necessary to develop techniques [13] that will allow the rapid and inexpensive determination of the most common *Salmonella* serotypes. Such methods [14, 15] can be incorporated into easy-to-use formats [8, 13, 16] that will be acceptable to primary laboratories.

The genes required for synthesis of the O antigen in *S. enterica* strains are found in a cluster between the *galF* and *gnd* genes on the bacterial chromosome [17]. Protein products of the genes within these clusters can be generally divided into three groups: (i) those required for the synthesis of the sugars, (ii) those involved in the transfer and modification of the O units, and (iii) those necessary for the polymerization and transport of the O units [17]. While the sugar biosynthetic genes have been found to be quite homogeneous between *S. enterica* strains, the transferase/flippase and polymerization genes encoded by the *wzx* and *wzy* genes show a great deal of heterogeneity. This heterogeneity can be used as the basis for the development of novel molecular serotyping methods [16]. 

We have sequenced the O antigen gene cluster of two *S*. Pomona O28 isolates and a *S*. Dakar O28 reference strain. Despite the fact that *S*. Dakar and *S*. Pomona have the same O serotype, the O antigen gene cluster of *S*. Dakar was quite different than that of *S*. Pomona. These results suggest that the O antigen oligosaccharides of *S*. Pomona O28 and *S*. Dakar O28, though they contain at least one common epitope, may be structurally different.

## 2. Materials and Methods

### 2.1. Salmonella Isolates

*Salmonella enterica* subsp.* enterica* serotype Pomona (serotype 28_1_28_2_:y:7; NML number 07-0213) was from the strain collection of the OIE Salmonella Reference Laboratory at the Laboratory for Foodborne Zoonoses, Guelph, ON. The *S*. Pomona reference strain S-1467 (28_1_28_2_:y;1,7) and *S*. Dakar strain S-1097 (28_1_, 28_3_:a:1,6) were from the Enterics reference strain collection at the National Microbiology Laboratory (NML), Winnipeg, MB. Strain S-1467 was originally obtained from the Institut Pasteur in 1999 while S-1097 is a culture type strain obtained in 1972 from the Public Health Laboratory Service in Colindale, UK (their strain number JT 987). 

### 2.2. Amplification of the O-Antigen Gene Cluster

The O-antigen gene cluster between the JUMPStart sequence [18] and *gnd* from isolate 07-0213 was amplified by long range PCR using primers 412 and 482 [19] with an Expand Long Range dNTPack kit (Roche Diagnostics, Laval, QC, Canada) following to the methods of the manufacturer. Template DNA was prepared using the protocol of [20]. The amount of DMSO used in each PCR reaction was optimized to 5% (vol/vol). The amplification conditions were 92°C for 2 minutes; 10 cycles of 92°C for 10 seconds, 65°C for 15 seconds, and 68°C for 15 minutes; 20 cycles of 92°C for 10 seconds, 65°C for 15 seconds, and 68°C for 15 minutes plus 20 seconds added to each additional cycle; and final extension at 68°C for 7 minutes. Following PCR amplification, amplicons were visualized on 1% agarose (Invitrogen Canada, Burlington, ON, Canada) gels after staining with ethidium bromide.

### 2.3. Cloning of the O-Antigen Gene Cluster DNA

Amplicons from several PCR reactions were pooled and sheared in a nebulizer (Invitrogen) for 3 minutes at 20 psi to obtain fragments between 0.5 and 4 kb. The pooled fragments were purified using Montage PCR Centrifugal Filter Devices (Millipore, Billerica, MA, USA) and cloned into the pCR4-TOPO vector using the TOPO TA Cloning kit as instructed by the manufacturer (Invitrogen). Transformants of *E. coli* strain DH5*α* were selected on Luria-Bertani agar plates containing 100 *μ*g mL^−1^ ampicillin with added X-Gal-IPTG (40 *μ*g mL^−1^; USB Corporation, Cleveland, OH, USA). DNA was isolated from positive (white) clones by the boiling technique [21]. 

### 2.4. Sequencing of the O-Antigen Cluster DNA

The *Salmonella* DNA inserts were amplified in PCR reactions using the FastStart Taq DNA polymerase kit (Roche Diagnostics Laval QC Canada) with primers M13 (5′-GTAAAACGACGGCCAGT-3′) and T7 (5′-GTAATACGACTCACTATAG-3′) complementary to specific plasmid sequences flanking the insertion site. Amplification conditions were 94°C for 5 minutes, 35 cycles of 94°C for 30 seconds, 50°C for 30 seconds, and 72°C for 45 seconds, followed by a final extension at 72°C for 5 minutes. Amplicons were visualized on agarose gels as above, purified by the DNA Core Facility at the National Microbiology Laboratory using the Agencourt Ampure PCR purification system (Agencourt Bioscience Corp., Beverly MA, USA), and sequenced using the M13 and T7 primers. DNA sequencing was performed by the DNA Core Facility at the National Microbiology Laboratory using Big Dye Terminator 3.1 Cycle Sequencing kits (Applied Biosystems, Foster City, CA USA) according to the manufacturer's instructions. DNA sequence data was generated using either an ABI 3100 or 3730 DNA Analyzer (Applied Biosystems). Lasergene DNASTAR software (DNASTAR Inc., Madison WI USA), Kodon (Applied Maths, Austin, TX) and Psi-BLAST (http://www.ncbi.nlm.nih.gov/blast/Blast.cgi) were used for editing, assembling, and annotation of DNA sequences. 

### 2.5. GenBank Submission

The sequences described in this manuscript were deposited with GenBank under accession numbers EU805803 (*S*. Pomona NML 07-0213) and FJ467642 (*S*.Dakar S-1097).

## 3. Results

**Figure 1:**
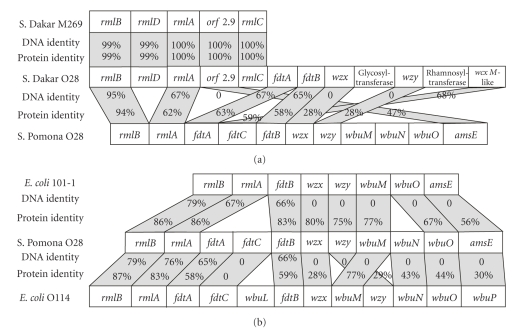
Comparison of O antigen regions: A. *S. *Dakar O28 versus *S. *Pomona O28 and *S. enterica* serotype Dakar M269; B. *E. coli* 101-1 versus *S. *Pomona O28 and *E. coli* O114. The *S*. Pomona and *S*. Dakar O-antigen cluster DNA sequences were deposited with GenBank (accession numbers EU805803 and FJ467642, resp.). The *E. coli* O114 O antigen cluster was accession number AY573377, while *E. coli *101-1 O antigen cluster sequence data was from *E. coli* 101-1 gcontig_1112603666495, accession number NZ_AAMK02000002.1.

Long PCR amplification of DNA from strain *S*. Dakar S-1097 using JUMPstart and *gnd *primers produced a product of 11,386 bp (Table 1). The *S*. Pomona O28 isolate 07-0213 O antigen gene cluster was 10,125 bp long and contained 11 open reading frames (Table 2). The sequence of this region from reference strain *S*. Pomona isolate S-1467 was also determined and found to be identical to the 07-0213 sequence from nucleotides 50–10,010 (data not shown). All O antigen cluster ORFs had low %G + C content and significant homology to genes from several other bacteria (Tables 1 and 2, Figure 1). 

The gene order of the *S*. Dakar O28 O antigen gene cluster was very different than that of *S*. Pomona O28 (Figure 1, compare Tables 1 and 2). The first five genes of the *S*. Pomona O28 cluster were *rmlB*, *rmlD*, *rmlA*, *orf2.9*, and *rmlC* (Table 2), confirming the results previously reported by Li and Reeves [19] for the partial O antigen gene cluster sequence of a *S*. Dakar isolate. Of these, only *rmlA* and *rmlB* were present in *S*. Pomona; *orf2.9* was moderately homologous to the *amsE* homolog of *S*. Pomona (Figure 1). The *wcxM*-like gene of *S*. Dakar S-1097 was in the opposite orientation with respect to the rest of the O antigen cluster, suggesting an independent origin. This gene was homologous to the *fdtC* gene of S. Pomona strain 07-0213 (Figure 1). Identification of the *S*. Pomona *rmlA* and *rmlB* genes was unambiguous, though *rmlA* had only moderate homology with *rmlA* genes from other *Salmonella* and *E. coli*. The 42.9% G + C content of *rmlB* from the *S*. Pomona O28 isolate was close to the average of this gene in other *Salmonella* isolates [19]. On the other hand, the *S*. Pomona *rmlA* %G + C content of 37.4% was much lower than that found in other *Salmonella* O antigen sequences. 

The *S*. Pomona O antigen gene cluster showed similar organization to similar clusters from two *E. coli* strains (Figure 1). The* rmlA* and* rmlB* genes showed significant identity among all three strains. While both the *fdtA* and *fdtC* genes were similarly located in the O antigen clusters of *S*. Pomona and *E. coli* O114, only the* fdtA* gene/FdtA protein exhibited significant sequence identity (Figure 1). Both genes were absent from *E. coli* 101-1. Though the* fdtB* genes of both *E. coli* strains shared a similar degree of identity with *fdtB* from *S*. Pomona, the Fdt protein from *E. coli* 101-1 had a higher level of identity with the *S*. Pomona protein. All remaining genes of *S*. Pomona had no identity with the corresponding *E. coli* gene, though there were high levels of identity of the translated proteins (Figure 1). 

The *wzx* and *wzy* genes were identified on the basis of homology of the translated protein with other genes (Tables 1 and 2, Figure 1). The topology of the translated protein products of these genes was determined to ensure that it was consistent with the proposed designation. The predicted transmembrane structure of Wzx and Wzy was confirmed using the TMHMM Server v. 2.0 (http://www.cbs.dtu.dk/services/TMHMM) and the HMMTOP (http://www.enzim.hu/hmmtop/) servers, with the *wzx* translation product having 12 predicted membrane-spanning regions and the *wzy* translation products having 10. Both the *wzx* and *wzy* genes of *S*. Dakar O28 were unique and very different from the *wzx *and *wzy* genes of *S*. Pomona; the proteins showed only 28% identity (Figure 1). The *S*. Pomona Wzx and Wzy proteins had strong identity with their homologs in *E. coli* 101-1 and lower identity with Wzx and Wzy from *E. coli* O114 (Table 2, Figure 1). In both cases there was no identity at the DNA level, indicating convergent evolution of the protein without transfer of genes between either *E. coli* strain and *S*. Pomona.

Serotyping of the *Salmonella *and *E. coli* strains was performed by bacterial agglutination assays by the Identification and Serotyping Section, National Microbiology Laboratory, using *Salmonella* and *E. coli* specific rabbit antisera. These antisera were prepared, absorbed where necessary, and subject to stringent quality control by the NML according to reference methods [1, 3, 24]. *Salmonella* O antigens were determined by slide agglutination, whereas *Salmonella* H antigens and *E.coli* O and H antigens were determined by tube agglutination. In slide agglutinations the *S*. Dakar O28 reference strain S-1097, the *S*. Pomona O28 07-0213 strain, and the *S*. Pomona reference culture (S-1467) all gave a 4+ reaction when tested with anti-O28 antiserum. An *E. coli* O114 reference culture (EC 200) tested with the same antiserum resulted in a negative reaction.

## 4. Discussion

**Figure 2:**
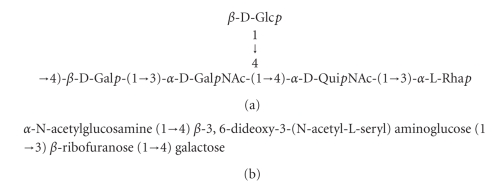
Oligosaccharide structures of known *Salmonella* and *E. coli* O antigens: A. *Salmonella* Dakar O28 O antigen structure [22] and B. *E. coli *O114 O-specific polysaccharide [23]. Gal: galactose, Glc: glucose, GalNAc: N-acetylgalactosamine, QuiNAc: N-acetylquinovosamine (N-acetyl-2-amino-2,6-dideoxy glucose); Rha: rhamnose.

The DNA sequence of the *S*. Dakar O antigen gene cluster is consistent with the known structure of its O antigen oligosaccharide (Figure 2). Rhamnose is produced by the *rmlA*, *B*, *C*, and *D* gene cluster [17] and the O antigen oligosaccharide of *S*. Dakar contains rhamnose (Figure 2). A putative rhamnosyltransferase was also identified in the *S*. Dakar O antigen gene cluster (*orf11* in Table 1). Though *rmlB* and *rmlA* were present in both *S*. Pomona and *S*. Dakar, they were closest in homology to proteins from different sources (compare Tables 1 and 2), suggesting that they may have been acquired from different sources. The *S*. Pomona O28 O antigen cluster did not contain the *rmlC* and *rmlD* genes necessary for production of rhamnose (Figure 1, Table 2). Furthermore, none of the other genes that were present would be expected to be active in the synthesis of this 6-deoxy-hexose [17]. This differential production of rhamnose must be confirmed by structural studies of the *S*. Pomona O antigen oligosaccharide. If true, it could contribute to the known heterogeneity of O28 antigens. *Salmonella* serogroup O28 was originally divided into three subfactors—O28_1_, O28_2_, and O28_3_—without structural differences being ascribed [22, 25, 26]. *S*. Dakar expresses subfactors O28_1_ and O28_3_, whereas subfactors O28_1_ and O28_2_ are present in the LPS of *S*. Tel-Aviv and *S*. Pomona. 

*fdtA* (dTDP-6-deoxy-3,4-keto-hexulose isomerase) and *fdtB* (dTDP-6-deoxy-D-xylo-hex-3-ulose aminase) genes of were identified in *S*. Pomona and *S*. Dakar. A homolog of the *fdtC* (putative acetylase) gene was identified in *S*. Pomona, which and analysis suggested is a WcxM-like protein. We suggest that the gene was indeed *fdtC* based on two pieces of evidence: (1) a *fdtC* gene is present at the same location in the *E. coli* O114 O antigen gene cluster, and (2) *fdtA*, *fdtB*, and *fdtC* together comprise a functional unit [17]. A putative gene (*orf12* in Table 2), also encoding a WcxM-like protein, was found in the *S*. Dakar O antigen gene cluster. This gene would appear to be a homolog of the *fdtC* gene of *S*. Pomona and *E. coli* O114. Since the *S*. Dakar* fdtC* homolog is present in the reverse orientation compared with other genes of the O antigen cluster, it has been acquired independently of these other genes. Its position at the end of the gene cluster differs markedly from the position of *fdtC* in *S*. Pomona (Figure 1). 

The *rmlA* and *rmlB* genes encode the first two enzymes of the rhamnose biosynthetic pathway in *Salmonella* and *E. coli* [17, 19]. Beginning with glucose-1-phosphate, these two genes produce dTDP-6-deoxy-D-*xylo*-4-hexulose. This intermediate can then be converted to 3-acetamido-3,6-dideoxy-D-galactose by the *fdtA*, *fdtB*, and *fdtC* genes [17]. The *fdtC* gene was a homolog of *wxcM* genes that encode bifunctional enzymes, in which the amino terminal part of the proteins is homologous to acetyltransferases and the carboxy terminal portions are similar to isomerases responsible for isomerisation of 4-keto hexoses to 3-keto hexoses. If both activities are indeed functional in the *S.* Pomona FdtC protein, this protein could be responsible for the production of the Quip3NAc sugar (Figure 2; [17]) that is known to be present in the *S*. Dakar O28 O antigen [22] and further suggests that the sugar may be present in *S*. Pomona. Alternatively, *S*. Pomona may indeed incorporate 3-amino-3,6-dideoxy-D-galactose into its O antigen oligosaccharide. Structural determinations are required to resolve this question. *E. coli* O114 strain E2808 contains in its O-antigen a sugar very closely related to Quip3NAc, namely, 3,6-dideoxy-3-(*N*-acetyl-L-seryl)-aminoglucose [23]. The precursor of this sugar is likely the product of those genes homologous to the *S*. Pomona O28 genes that as we suggest may be implicated in 3-amino-3,6-dideoxy-D-galactose and/or Quip3NAc synthesis.

*S*. Dakar *orf9* (Table 2) putatively encodes a protein having a very low homology to members of the glycosylase 2 family that was not found in *S*. Pomona. This strongly suggests that the oligosaccharide produced by *S*. Dakar will differ from that produced by *S*. Pomona. The product of the fourth open reading frame (*orf2.9*) was also a putative glycosyltransferase [19]. Together, these proteins would likely be responsible for adding two or more of the remaining three sugars to the *S*. Dakar O28 O antigen oligosaccharide (Figure 2). *S*. Pomona ORFs annotated here as *wbuM* and *amsE* also had strong homology with proteins belonging to the glycosyltransferase 2 family, though the specific function of these transferases cannot be inferred from DNA sequence alone [17]. There was no significant identity at either the DNA or protein level between these glycosyltransferases of *S*. Dakar and *S*. Pomona, suggesting that the O antigen oligosaccharides of these two isolates may contain further differences. 

The protein encoded by the ORF designated *wbuO* contained no known conserved domains, and the function of the *E. coli* homolog has not been determined. Two other homologs (ACK44395 and ACD75797), which contain eight transmembrane domains, are designated as O-antigen acyltransferases. 

Overall, the *S*. Pomona O28 O antigen gene cluster showed a remarkable conservation of gene order with the O antigen gene clusters from *E. coli* 101-1 and *E. coli* O114:H32 type strain G1088 (Figure 1, [27]). No structural analysis for the *E. coli* 101-1 O antigen polysaccharide was found. The *E. coli *O114 O-antigen oligosaccharide (Figure 2) consists of equimolar amounts of galactose, ribose, N-acetylglucosamine, and 3,6-dideoxy-3-aminoglucose [23]. This is consistent with a role for the conserved *wbuM*, *-N*, and *-O* genes in transfer of galactose and glucose (or N-acetylglucosamine) to the O-antigen oligosaccharide and a role for the genes unique to *E. coli* O114 in the transfer of ribose. Ribose would therefore not be expected to be part of the *S*. Pomona O28 O antigen oligosaccharide. An additional gene (*wbuL*) was present in the *E. coli* O114 strain immediately downstream of the *fdtC* gene but was absent in *S*. Pomona. The final gene in the *S*. Pomona O antigen cluster showed a higher homology with *wbeD* from *E. coli* O117 [28] than that with the *wbuP* gene from *E. coli* O114. 

The *wbuL* and *wbuP* genes of *E. coli* O114 are both glycosyltransferases that have no homolog in *S*. Pomona O28; these two genes may alter the *E. coli* O antigen structure in a fashion that either does not allow it to be recognized by antiserum against the *Salmonella* O28 serogroup or creates an alternative immunodominant epitope. This view is supported by the low homology of the *wzy* gene from *S*. Pomona O28 with the *wzy* gene from *E. coli* O114 and by the fact that the *S*. Pomona *wzx* gene was most closely homologous to a gene from *Geobacter metallireducens* GS-15.

The *S*. Pomona O antigen cluster could have been assembled using the *wbuM*, *wbuN*, and *wbuO* genes, at least, from *E. coli* O114 and other genes from a variety of different sources. *E. coli* O103 isolate H515b (GenBank accession numbers AY532664 and EF027106) has in its O antigen gene cluster homologs of the *S. *Pomona O28 *rmlB*, *rmlA*, *fdtA* (*wbtA*), *fdtC* (*wbtB*), and *fdtB* (*wbtC*) genes in the same order preceding *wzx* [29]. *E. coli* H515b strain, or a similar serogroup O103 *E. coli*, could also have been the source of the first five genes of the *S. *Pomona O28 O antigen gene cluster. 

Information provided through sequencing of the *S*. Pomona and *S*. Dakar O-antigen gene clusters will allow probes for the various O28 *wzx* and *wzy* genes to be included in updated versions of DNA microarray-based *Salmonella* serotyping assays, as well as in assays comprising other formats [30]. This will make accurate serotyping more accessible for primary laboratories within the health care system. 

Differences in both the O antigen gene organization and content, as well as in the *wzx* and *wzy* gene sequences, suggest that the O antigen oligosaccharides of *S*. Dakar and *S*. Pomona may each have a different chemical structure but that both fortuitously contain the dominant O28 epitope. This needs confirmation in structural studies that are beyond the scope of the current investigations. Whether this situation occurs in any other serogroup is not known. It is clear that the development of molecular serotyping methods and the interpretation of results from these methods will require characterization of the relevant genes from each *Salmonella* serotype. Furthermore, the data presented here reinforce the observation that two isolates with the same serogroup may not, in fact, have the same gene content. Interpretation of the meaning of serovar identity and its relationship with virulence and host restriction or adaptation then becomes somewhat more problematic. For some purposes it may be of greater advantage to determine the genovar [11] of an isolate.

## Figures and Tables

**Table 1:** Putative genes comprising the O antigen cluster of *S.* Dakar isolate S-1097.

ORF	Position in Sequence (nt)	Length (bp)	%G + C	% DNA identity	Species with the closest DNA identity	Accession No.	Protein length (aa)	Similar protein	Protein Function	Species with closest protein homolog	% amino acid identity	Accession No. of closest homolog
1	105–1190	1086	43.46	99%	*S. enterica* M269	AF279620.1	361	RmlB	dTDP-glucose 4,6 dehydratase	*S. enterica* serotype Dakar M269	99%	AAG09517.1
2	1190–2089	900	50.00	99%	*S. enterica* M269	AF279620.1	299	RmlD	dTDP-4-dehydrorhamnose reductase	*S. enterica* serotype Dakar M269	99%	AAG09518.1
3	2137–3009	873	43.34	100%	*S. enterica* M269	AF279620.1	290	RmlA	D-glucose-1-phosphate thymidylyltransferase	*S. enterica* serotype Dakar M269	100%	AAG09519.1
4	3038–3853	816	35.17	100%	*S. enterica* M269	AF279620.1	271	Orf 2.9	glycosyltransferase	*S. enterica* serotype Dakar M269	100%	AAG09520.1
5	3884–4435	552	33.70	100%	*S. enterica* M269	AF279620.1	183	RmlC	dTDP-6-deoxy-D-glucose-3,5-epimerase	*S. enterica* serotype Dakar M269	100%	AAG09521.1
6	4428–4829	403	32.09	71%	*Pectobacterium carotovorum* subsp. *carotovorumI *PC1	CP001657.1	133	FdtA	dTDP-6-deoxy-3,4-keto-hexulose isomerase	*Pectobacterium carotovorum* subsp. *carotovorum* PC1	73%	YP_003016883.1
7	4859–5962	1104	33.42	67%	*Photorhabdus luminescens* subsp. *laumondii *TTO1	BX571875.1	367	FdtB	aminotransferase	*Vibrio harveyi* HY01	61%	ZP_01987033.1
8	5959–7224	1266	32.94	73% over 169 nt	*Enterobacter sakazakii* strain NCTC 11467	EU076545.1	421	Wzx	O-antigen flippase	*Pectobacterium carotovorum* subsp. *carotovorum* WPP14	48%	ZP_03830717.1
9	7231–8637	1407	30.14	—	none	—	468	putative glycosyltransferase	glycosyltransferase	*Francisella philomiragia* subsp. *philomiragia* ATCC 25015	33%	ZP_04755635.1
10	8637–9932	1296	30.32	—	none	—	431	Wzy	oligosaccharide repeat unit polymerase	*Ralstonia pickettii* 12J	29%	YP_001898213.1
11	9922–10749	828	34.06	—	none	—	275	glycosyltransferase family 2	rhamnosyltransferase	*E. coli *ATCC 8739	32%	YP_001724596.1
12	11222–10752	471	35.88	70%	*Shewanella denitrificans* OS217		156	WcxM-like protein	acetyltransferase	* Providencia alcalifaciens *DSM 30120	69%	ZP_03320663.1

Nt: nucleotides; bp: base pairs; aa: amino acids.

**Table 2:** Putative genes and proteins comprising the O antigen cluster of *S.* Pomona isolate 07-0213.

ORF	Position in Sequence (nt)	Length (bp)	%G + C	% DNA identity	Species with the closest DNA identity	Accession No.	Protein length (aa)	Similar protein	Protein Function	Species with closest protein homolog	% amino acid identity	Accession No. of closest homolog
1	101–1174	1074	42.92	97	*S*. Paratyphi A AKU_12601	FM200053.1	357	RmlB	dTDP-glucose 4,6 dehydratase	*S*. Typhi CT18	96	NP_456646.1
2	1176–2039	864	37.38	76	*E. coli* O103:H2 strain 12009	AP010958.1	287	RmlA	D-glucose-1-phosphate thymidylyltransferase	*E. coli *101-1	86	ZP_03068444.1
3	2043–2438	396	33.08	71	*E. coli* serogroup O91	AY035396.1	131	FdtA	dTDP-6-deoxy-3,4-keto-hexulose isomerase	*Pectobacterium carotovorum *subsp.* brasiliensis *PBR1692	70	ZP_03826801.1
4	2435–2902	466	35.68	69	*E. coli* serogroup O2	EU549863.1	154	FdtC	acetyltransferase	*Providencia rettgeri* DSM 1131	68	ZP_06127571.1
5	2899–4002	1105	36.05	68	*E. coli* strain H515b	EF027106.1	368	FdtB	aminotransferase	*E. coli *101-1	83	ZP_03068415.1
6	4005–5261	1257	30.15	—	none	—	418	Wzx	O-antigen flippase	*E. coli *101-1	80	ZP_03068086.1
7	5262–6578	1317	29.31	—	none	—	438	Wzy	oligosaccharide repeat unit polymerase	*E. coli *101-1	75	ZP_03068123.1
8	6565–7440	876	29.79	71% over 122 nt	*Pasteurella multocida* subsp. *multocida* strain Pm70	AE004439.1	292	WbuM	glycosyltransferase group 2 family	*E. coli *101-1	77	ZP_03068240.1
9	7440–8123	684	29.09	—	none	—	227	WbuN	phosphoserine phosphatase	*E. coli* O114	43	AAT77178.1
10	8110–8901	792	26.44	—	none	—	263	WbuO	O-antigen acyltransferase	*E. coli *101-1	67	ZP_03068384.1
11	8894–9718	825	34.18	74 (128 nt)	*E. coli* O117	EU694096.1	274	AmsE	amylovoran biosynthesis glycosyltransferase	*E. coli *101-1	56	ZP_03068341.1
